# Hustle: Experiences of making work ‘work’ for non-standard and precariously employed workers in NYC: Isabel Cuervo

**DOI:** 10.1093/eurpub/ckac130.124

**Published:** 2022-10-25

**Authors:** I Cuervo, EQ Ahonen, EF Vignola, L Davis, S Baron

**Affiliations:** 1 Commoner Center Health and Environment, Queens College, City University of New York, Flushing, USA; 2 Occupational and Environmental Health, University of Utah School of Medicine, Salt Lake City, USA; 3 Community Health and Social Sciences, CUNY Graduate School of Public Health, New York, USA; 4 Boston, USA

## Abstract

**Background:**

Precarious and non-standard employment (NSE) has negative implications for workers’ health. As part of a six-country comparative mixed methods case study, this research explores US-based workers’ experiences in NSE and its influences on their health and well-being in a context of weak labor regulations and social welfare programs.

**Methods:**

To understand US policy context, we analyzed country-level labor regulatory and social protection frameworks using 2019 Organization for Economic Cooperation and Development data. To understand workers’ experiences, we conducted in-depth interviews with NSE workers in various occupations in New York City (N = 40) between January and May 2021. We recruited and screened eligibility via Facebook advertisements and an online questionnaire, respectively. We used deductive and inductive thematic analysis for interview data.

**Results:**

With heavy reliance on market competition in the US, minimal state regulation and flexible labor markets create less secure employment along with limited government-funded social supports. Workers’ experiences center on the Hustle, i.e., figuring out how to make NSE work for them and their families. They lack healthcare coverage and have low expectations of other supportive employment and social protections (e.g., paid leave). While NSE payoffs (e.g., perceived flexibility) were common for most, almost all experience NSE tradeoffs (e.g., job insecurity and instability) that create stress and overwork, negatively implicating overall health and well-being. These impacts differ by access to resources associated with social location (e.g., immigration status). COVID-19 exacerbated these experiences.

**Discussion:**

Low expectations of supportive policies of US workers in NSE are linked to the individualized hustle, as they attempt to counter NSE tradeoffs often relying on family to fill those gaps. Over-reliance on privatization for social supports such as healthcare coverage can be detrimental to workers’ health.

**Key messages:**

- US workers in NSE experience stress and overwork with low expectations of support from the state.
- We caution against increasingly market-based policies in Europe, which may jeopardize public health.

